# Editorial: Global occurrence of pine wilt disease: Biological interactions and integrated management

**DOI:** 10.3389/fpls.2022.993482

**Published:** 2022-07-26

**Authors:** Margarida Espada, Anna Filipiak, Hongmei Li, Ryoji Shinya, Claudia S. L. Vicente

**Affiliations:** ^1^MED—Mediterranean Institute for Agriculture, Environment and Development & CHANGE—Global Change and Sustainability Institute, Institute for Advanced Studies and Research, Universidade de Évora, Pólo da Mitra, Évora, Portugal; ^2^Institute of Plant Protection—National Research Institute, Poznań, Poland; ^3^Department of Plant Pathology, Nanjing Agricultural University, Nanjing, China; ^4^School of Agriculture, Meiji University, Kawasaki, Japan

**Keywords:** tree pathogen, control, plant-parasitic nematode, pinewood nematode, plant nematode interaction, forestry sustainable management

Plant pathogens cause severe losses in a wide range of crops and forestry plant species worldwide, being a major obstacle toward achieving sustainable agriculture and forestry. In forests, pathogens can affect sustainable management by affecting economic trade and serious ecological losses can occur, such as the ability to store carbon, reduce flood risk or purify water (Boyd et al., 2013). Ranking in the top ten of the most damaging plant-parasitic nematodes worldwide, the migratory endoparasitic nematode *Bursaphelenchus xylophilus* (pinewood nematode, PWN) is the causal agent of Pine wilt disease (PWD) being responsible for the tremendous decline of conifers species in Eurasian conifer forests (Mota and Vieira, 2008; Futai, 2013; Jones et al., 2013). This complex disease results from a tripartite species interaction (plant-nematode-insect), where each participant involved may be a target for research and understanding at a molecular, evolutionary, chemical, and biological levels. In the present climate change and global warming scenarios, the dispersal of this pathogen has negative impact in forestry practices. The PWN pathogenicity is more severe at high temperatures and drought will likely emphasize PWN-associated issues, increasing its adaptability and incidence on conifers forest worldwide (Ohsawa and Akiba, 2014; Hirata et al., 2017). Currently, the strategies used for PWN control are preventive and new durable and greener solutions are needed. This Research Topic gathered work on several aspects of this disease aiming for a better understanding not only on the molecular mechanisms by which PWN interacts with all partners in the disease, but also in the development of new control targets and methods that could be potentially used in plant science diagnostic and research. Here, we compiled eleven original research articles and two theoretical issues that focus on different aspects of the complex PWD. An overview of the scientific content is summarized below.

Pest management strategies relies on efficient detection methods (Barzman et al., 2015). The close morphological similarity of *B. xylophilus* to a number of other tree-inhabiting species from the *xylophilus* group makes the problem particularly important, as any misidentification of nematodes extracted from the material subjected to phytosanitary inspection may lead to disturbances in the international wood trade and serious economic consequences (Braasch et al., 2001). For the development of new PWN detection strategies, two new molecular alternatives were proposed. Zhou et al. developed a rapid detection system for *B. xylophilus* that allow non-nematology researchers to detect the presence of PWN, as well as other nematodes, by RPA-LFD (recombinase polymerase amplification combined with lateral flow dipstick) in biological material. Based in previous studies (Meng et al., 2018), Meng et al. proposed a new LAMP methodology for detection and identification of pine wood nematode targeting the possibly pathogenicity-related gene. This technique can be used as a routine and/or combined with traditional PCR.

The complexity of this disease is also a result of the biological interactions co-occurring between plant-nematode-microbe. Tian et al. studied the temporal variation of bacteria to fungi ratio in PWD sites with different PWN invasion histories and evaluated the insecticidal/nematicidal activities of the most abundant bacteria (genus *Serratia*) extensively found in the pupal chambers and the tracheae of the *Monochamus alternatus*. The authors found that microbial communities changed with the duration of PWN invasion, and that *Serratia* were more expressive in sites with the longest PWN presence. In the same line of thought, Vicente et al. showed that fungal communities also changed along sites with different PWD history, emphasizing the effect of a prolong exposure to PWN has a tremendous effect on these co-occurring multi-species interactions.

Understanding the molecular mechanisms underpinning the parasitism of the PWN are pivotal for the development of novel control strategies. Since the release of genomic and transcriptomic data there has been an effort to study potential parasitism-related genes and understand their evolutionary and functional role in plant-parasitic nematodes. Ning et al. presented a study on comparison of C-type lectin (CTL) family genes in insect-vectored and self-dispersing nematodes, and demonstrated that this gene family contributed to this evolutionary transition. In the phoretic nematode *B. xylophilus*, the similarity in the CTL gene family sizes, that clustered into separate phylogenetic *sister* clades, is due to parallel evolution, when similar phenotypes occur in closely related taxa, that are produced by orthologous genes. In a functional study, Wang et al. showed that PWN UGTs (uridine diphosphate-glycosyltransferases), specifically the UGT440A1, may be involved in the pathogenic process of the PWN in the disease. Knocking down UGT440A1 suppressed PWN core metabolism (motility, feeding and reproduction) which *in planta* slowed down their infectiveness. In a different approach, Cardoso et al. presented a comparative proteomic study in two PWN isolates with different virulence and geographical location. Thirteen proteins were found significantly increased in the proteome of the virulent isolate as compared with the less virulent one and were selected as potential virulence biomarkers. One of the main research challenges to study tree pathogens is the lack of genetic tools to functionally analyze nematode parasitism candidate genes. Kirino et al. described a new powerful tool for transient overexpression of nematode genes using an exogenous gene expression system applied in pine seed embryos with ALSV vector (virus vector). These authors showed that the use of natural host pine embryos is more reliable for functional analysis than using *Nicotiana benthamiana*-based methods.

The basis of the pine host genetic traits for resistance to PWD will be relevant to the future pine breeding programmes. Hirao et al. focused on the genetic mapping of resistance to PWD with the goal of improving resistance in Japanese Black Pine (*Pinus thunbergii*). The authors provided a high-density linkage map and quantitative trait loci (QTL) mapping of PWD resistance for *P. thunbergii*. This study also validates the GBS technology and UNEAK pipeline as tools for use in QTL mapping in the species without a reference genome sequence. The results demonstrated that the locus linked to PWD resistance was identified on LG-3. This locus will be a good candidate to develop molecular markers associated with the phenotype. In Europe, maritime pine (*Pinus pinaster*) is the one of most susceptible hosts to PWN and genetic variability has been found in natural populations associated to heritable PWD resistance. Rodrigues et al. presented a physiologic and primary metabolite responses of susceptible and resistance *P. pinaster* seedlings to PWN. The authors showed that specific traits are associated with pine resistance to PWD, such as GABA shunt-related metabolites, and the plant defense response was regulated and able to reduce the ability of the nematode migration. Jeon et al. tested previously known 12 SAR (systemic acquired resistance) elicitors to manage PWD by SAR in pine trees. Methyl salicylate (MeSA) was found to induce resistance against PWD in *Pinus densiflora* seedlings. Moreover, qRT-PCR analysis confirmed that MeSA induced the expression of defense-related genes, and can inhibit and delay the migration and reproduction of PWN in pine seedlings by modulating gene expression.

Forest mortality due to PWN infection, and the fast spread of PWD might be exacerbated by climate (more drought events and higher temperatures), topography, and human activities (i.e., infected timber exports). The study conducted by Estorinho et al. analyzed the symptom development of the PWD in three pine species inoculated with PWN under controlled temperature and water availability. The authors showed that the impact of PWN is species-dependent, being infected *P. pinaster* and *P. radiata* more prone to physiological and morphological damage than *P. pinea*. Moreover, they suggested a synergistic impact of PWN infection and drought triggering disease symptoms and mortality of these species. The spread of PWD in pine forests of the Mediterranean regions may be possibly facilitated by predicted drought conditions (i.e., high temperatures and low water availability). Xia et al. demonstrated a theoretical method based on the individual dispersal of the insect vector—pine sawyer beetle (*Monochamus* spp.)—to elucidate the dispersal of PWD and evaluated the control strategies applied for the eradication of the disease in Korea.

In summary, this Research Topic with thirteen scientific contributions consolidates and expands our knowledge regarding recent advances in pine wilt disease.

## Author contributions

All authors listed have made a substantial, direct, and intellectual contribution to the work and approved it for publication.

## Conflict of interest

The authors declare that the research was conducted in the absence of any commercial or financial relationships that could be construed as a potential conflict of interest.

## Publisher's note

All claims expressed in this article are solely those of the authors and do not necessarily represent those of their affiliated organizations, or those of the publisher, the editors and the reviewers. Any product that may be evaluated in this article, or claim that may be made by its manufacturer, is not guaranteed or endorsed by the publisher.
